# Patients’ Experiences of Using an eHealth Pain Management Intervention Combined With Psychomotor Physiotherapy: Qualitative Study

**DOI:** 10.2196/34458

**Published:** 2022-03-16

**Authors:** Anne-Grethe Eiken, Dag Ø Nordanger, Lise Solberg Nes, Cecilie Varsi

**Affiliations:** 1 Department of Nursing and Health Promotion Faculty of Health Sciences Oslo Metropolitan University Oslo Norway; 2 Department of Digital Health Research Division of Medicine Oslo University Hospital Oslo Norway; 3 Institute of Clinical Medicine Faculty of Medicine University of Oslo Oslo Norway; 4 Department of Psychiatry and Psychology College of Medicine and Science Mayo Clinic Rochester, MN United States; 5 Faculty of Health and Social Sciences University of South-Eastern Norway Drammen Norway

**Keywords:** chronic pain, psychomotor physiotherapy, EPIO, self-management, telemedicine, mHealth, mobile phone

## Abstract

**Background:**

Chronic pain is a major health challenge to those affected. Blended care with psychomotor physiotherapy (PMP) combined with eHealth self-management might be beneficial.

**Objective:**

This study aims to explore how patients with chronic pain experience the combination of PMP and the use of EPIO, an eHealth self-management intervention for chronic pain.

**Methods:**

Individual semistructured interviews were conducted with 5 adult patients with chronic pain (ie, participants) who used EPIO in combination with PMP over a period of 10 to 15 weeks. Interviews explored participants’ experiences using this treatment combination in relation to their pain and analyzed their experiences using systematic text condensation.

**Results:**

Participants described having benefited from using EPIO in combination with PMP in terms of increased awareness of bodily signals and how pain was related to stress and activity. They also described changes in the relationship to themselves in terms of increased self-acceptance, self-assertion, and hope and their relationship to their pain in terms of seeing pain as less harmful and engaging in more active coping strategies.

**Conclusions:**

Results indicate that a blended care approach combining eHealth self-management interventions such as EPIO with PMP may be of value to patients living with chronic pain.

**Trial Registration:**

ClinicalTrials.gov NCT03705104; https://clinicaltrials.gov/ct2/show/NCT03705104

## Introduction

### Background

As much as 30% of the Norwegian population reports living with chronic pain [1-3], defined as pain lasting for more than 3 months [4]. Pain is one of the most common reasons for why people seek health care [5,6], sick leave, and disability pensions [7-10], and is also associated with increased mortality [11].

Chronic pain is complex, with physical, social, and psychological aspects interacting and impacting individual experience and often carrying major consequences for those affected and their social network [1,12]. Commonly reported pain-related challenges include sleep disturbances [13,14], anxiety and depression [13,15,16], and impairments of daily activities [13,17] and quality of life [13,18,19].

In multidisciplinary chronic pain management, the focus is generally on maximizing function and quality of life and may include combinations of medication treatment, physical therapy, and additional self-management approaches such as working with one’s own thoughts, perceptions, attitudes, emotions, and activities [20,21].

Self-management focuses on supporting people to actively manage their own health and live as well as possible with their chronic condition [22]. This includes problem solving relevant for dealing with the consequences of the chronic condition and making necessary changes in daily life to provide improved quality of life [22]. In recent years, active self-management has been increasingly considered an important factor for pain management. There is consistent evidence that the use of active pain self-management strategies, such as maintaining activities despite pain, causes less pain-related disability than those using more passive approaches, such as trusting others [23], and recent clinical guidelines also recommend including self-management interventions in chronic pain treatment [20].

Self-management for people living with chronic pain (ie, actively managing their own health) can be affected by biomedical factors (eg, bodily symptoms) and the psychosocial aspects of pain (eg, emotional distress, self-efficacy, motivation for change, acceptance, worst-case thinking, fear of pain, helplessness, and barriers to pain management) [24-26].

Many patients living with chronic pain are offered psychomotor physiotherapy (PMP) [27]. PMP focuses on changing and facilitating insight and awareness for the patient to recognize and understand the connections between their daily life and their physical condition [27]. On the basis of the notion that the body and psyche are deeply interconnected, PMP treatment aims to raise awareness and change the states of tension in the body, with the intention of increasing the patient’s familiarity and contact with his or her own body.

Interventions with cognitive behavioral therapy (CBT) [28] and acceptance and commitment therapy (ACT) [29] may also support self-management of chronic pain. CBT centers around the impact and relationships among thoughts, feelings, and behaviors and involves techniques to challenge and change thoughts and behaviors [28]. CBT approaches to chronic pain management have been associated with improved self-management; reductions in pain, disability, and emotional distress [30,31]; and improvements in mood, coping, and social functioning [30]. ACT focuses more on acceptance of a situation or setting, commitment to change, and attention to helpful strategies, with the goal of increasing psychological flexibility (eg, ability to adapt to situations and demands) [29,32] and has been associated with improvements in pain acceptance or psychological flexibility, pain intensity, disability or physical functioning, depressive symptoms, and quality of life [33,34].

In recent years, there has been an increasing interest in, and development of, eHealth CBT- and ACT-based interventions for pain management. The delivery of such interventions is not dependent on access to professionals with pain management competence, treatment waiting lists, or travel distances and may be less associated with stigma than more traditional forms of psychological treatment [13,35]. Although some eHealth interventions have been associated with decreased pain intensity and improved function and quality of life [35,36], the field of eHealth pain management is still at an early stage and most available eHealth programs so far lack a theoretical foundation and have not been subjected to research and rigorous efficacy testing [36,37].

Studies have shown that blended care (eg, combination of therapy via eHealth and in-person care) [38] can provide new treatment possibilities and make it easier for patients to follow up on their own treatment between consultations [39]. An advantage of such blended care is that the health care provider and patient may plan the course of treatment together and that the provider may guide the patient when needed. As such, blended care may offer treatment that can be effective and, at the same time, cost-effective in terms of fewer health care sessions and may lower dropouts because of the individualized follow-up, and the continued access to the eHealth intervention may potentially also help maintain achieved long-term changes [40].

Patients with chronic pain and those providing treatment and care services to these patients have displayed an interest in, and a positive attitude toward, using eHealth self-management interventions for chronic pain [41,42]. Still, blended care in terms of PMP combined with eHealth self-management interventions is yet to be implemented and studied in clinical practice in Norway.

### Objectives

The aim of this study is to explore how patients with chronic pain experience the combination of PMP and the use of EPIO, an eHealth self-management intervention for chronic pain.

## Methods

### Study Design and Participants

This study is part of a larger project aiming to design, develop, pilot-test, and examine the effectiveness of EPIO, a digital self-management intervention for patients living with chronic pain in Norway. The design, development, and feasibility pilot-testing of EPIO have been published elsewhere [41-44], and a large-scale randomized controlled efficacy trial is currently ongoing.

This study adds to the larger project through reporting on qualitative findings from individual interviews conducted to explore patients’ experiences using EPIO in combination with PMP. The study was conducted at a physiotherapy institute in Norway. Recruitment processes and study execution was conducted by the first author, who is a psychomotor physiotherapist. To our knowledge, a few, if any, studies have examined delivery of blended care within PMP (eg, digital self-management in combination with PMP), and conducting a small-scale study was, therefore, considered appropriate to test delivery forms and study procedures. With options for the intervention to last up to 16 weeks (description of PMP and the EPIO intervention program is given later), good opportunities for in-depth descriptions from the participants regarding their experiences of using EPIO in combination with PMP were anticipated. Given the qualitative nature of the study, a predefined number of 6 participants representing patients living with chronic pain and receiving PMP was defined as acceptable (ie, minimum), as even a limited number of participants can allow for richness and depth in data related to participants’ experiences (ie, provided data collection methods).

Even with a small number of participants, the study aims for purposively sampling [45] to ensure some variations in gender and pain-related diagnoses. Participants were recruited between May and August 2019 and had to be aged ≥18 years, be able to understand and speak Norwegian, and had to have access to a smartphone or tablet with web access. Exclusion criteria were self-reported untreated severe mental illness, migraine, or cancer-related pain. Potential participants, already enrolled in a PMP treatment program at the physiotherapy institute, were identified based on the inclusion or exclusion criteria and informed about the study by the first author. As the first author was also the psychomotor physiotherapist of the potential participants, recruitment processes were carefully reviewed and discussed by the research team before recruitment to ensure attention to ethical considerations.

### The PMP Treatment Program

The PMP treatment program for people with chronic pain aims to facilitate change by raising insight into and awareness about the relationships between experiences and bodily pain [46]. Specifically, the psychomotor physiotherapist in this study worked to help facilitate changes related to bodily tensions, including aiming to strengthen coping mechanisms and reduce tension and pain in muscles, tendons, and joints. This was done by teaching specific movements and exercises and providing massages, all in parallel with seeking to increase patients’ knowledge and understanding related to physical reactions and the expression of such.

Part of the idea behind PMP is that people, often without real awareness of doing so, may use their posture, respiration, and muscles to *lock* feelings related to situations of conflict. By *pulling themselves together*, they may end up with tight muscles and subsequent related aches and pains [47]. In the PMP treatment program, the goal was to help raise insight into and awareness about these connections and reduce tension and facilitate change and ultimately to better understand potential *mind-body* connections so the patient could recognize their body as *one functional unit*.

### The EPIO Intervention Program

EPIO (inspired by the Greek goddess for the soothing of pain, Epione) is an app-based intervention program designed and developed by a collaborative research team consisting of scientists, health care providers, eHealth experts, content and system developers, and end user representatives [44]. The content is based on well-known, evidence-based aspects from CBT and ACT [28,29] and input from patients, spouses [41], and health care providers [42]. The intention of the EPIO program is to support self-management and coping for patients living with chronic pain.

**Figure 1:**
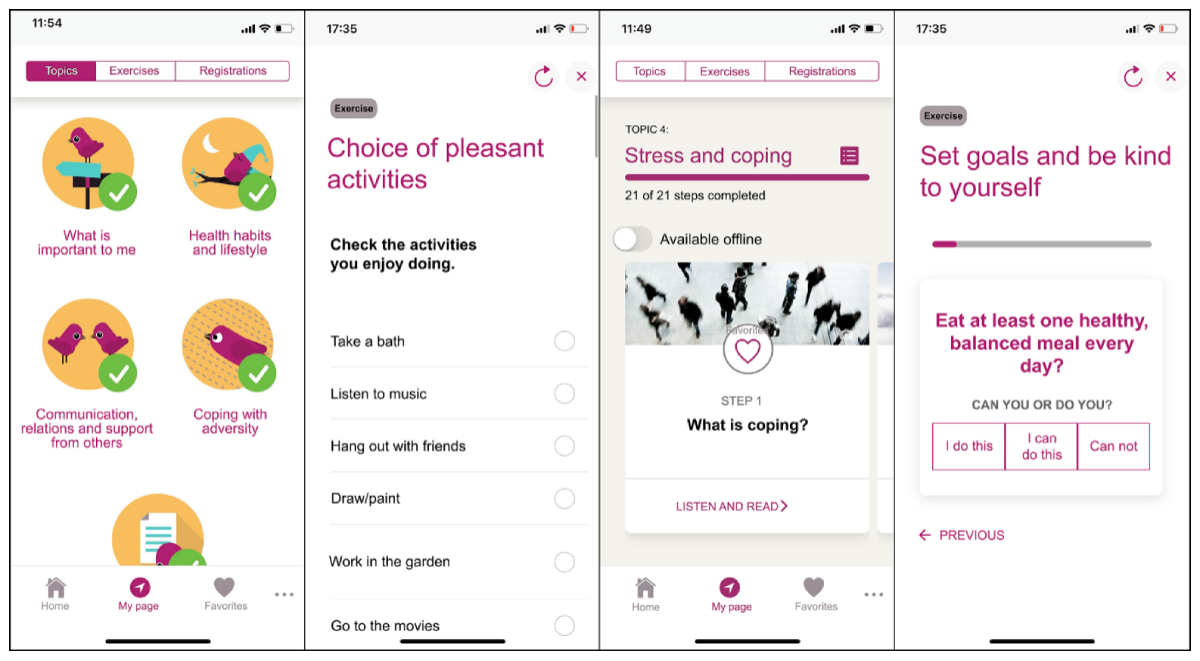
Example screenshots from the EPIO intervention program.

The EPIO program consists of 9 modules with informational and educational topics, including a variety of self-management–based coping techniques, such as breathing and relaxation exercises, cognitive restructuring exercises, and mindfulness-based information and training. The nine modules are (1) information about pain; (2) balance; (3) thoughts and feelings; (4) stress and coping; (5) what is important to me; (6) health behaviors and lifestyle; (7) communication, relation, and social support; (8) coping during difficult times; and (9) summary and the road ahead. Modules 1 to 5 must be opened in the presented order, whereas from 6 to 8, the user can choose what to open next. To encourage practice, each module must be completed and opened for 3 days before the next module can be opened. While working with the program, users can create their own favorite list by highlighting information and exercises they like, and they can also choose to receive reminders according to their own needs. Users can also choose between reading and listening to the program at any time. To ensure availability, the program can also be used in off-line mode. Screenshot examples from the EPIO intervention program are shown in Figure 1. The EPIO program has so far been tested in a feasibility pilot study with 50 participants, where the results showed EPIO to be perceived as useful and easy to use, with easily understandable content and excellent system usability [43].

When enrolled in the study, participants were introduced to the self-management program and received help downloading the EPIO app from App Store (Apple Inc) or Google Play Store, with instructions on how to get started. They were informed that they could use EPIO as much as they wanted in the study period of a maximum of 16 weeks. As such, they had access to a program containing some well-known but also many new exercises and aspects of information that they could use between PMP treatment sessions, potentially bringing new aspects into the treatment. When coming in for PMP treatment, the first part (ie, 5-10 minutes) of the face-to-face session with the psychomotor physiotherapist was spent talking about patients’ experiences of using EPIO since the last PMP session. They were given the opportunity to ask questions or introduce topics they had been involved in through EPIO or tell if there was something special that occupied them, thereby delivering EPIO in a blended care type of model.

### Data Collection Procedure

Information about age, diagnosis, and years of living with chronic pain was collected using a study-specific demographic form at study enrollment. Semistructured individual interviews were conducted following the completion of all modules in the EPIO program. A study-specific interview guide was developed based on literature examining and describing self-management of chronic pain, digital self-management interventions, and blended care health care delivery [4,24,27,30,35,36,39]. The interview guide contained questions about EPIO use and usefulness, pain impact, coping strategies, and potential experiences of change based on the use of EPIO (the interview guide is given in Multimedia Appendix 1). The questions were developed with the intention of allowing the participants to give a free description of their experiences. The interviews were conducted by the first author at her office from September to December 2019.

Each interview started with an open-ended question about the participant’s experience with using EPIO. On the basis of the answers, either new topics were addressed for follow-up or the next topic from the interview guide was introduced. The interviewer did not follow a specific order for the topics to be addressed but rather focused on having a fruitful conversation where topics were introduced and they appeared to fit naturally into the conversation, focusing on topics and hints addressed by the participant to capture issues raised. Furthermore, the interviewer asked clarifying questions to ensure that the essence of what the respondents described was clearly understood. The interviews lasted between 35 and 65 minutes, depending on the richness of the conversation. The interviews were audio recorded and transcribed verbatim, resulting in a total of 60 densely written text pages for analysis.

### Data Analysis

The transcripts were analyzed using systematic text condensation in 4 steps [48]. In step 1, the first author read through the transcripts several times while taking notes to get an overview of content and meaning. To strengthen the data analysis process, coauthors DN and CV also read through some of the transcripts to become familiarized with the material and prepare for further team analysis and discussions. In step 2, the first author identified meaningful entities (encoding) in collaboration with coauthors DN and CV and coded the identified material in the transcripts into 4 overarching themes using NVivo software (QSR International). In this process, the authors first identified preliminary themes, which were then discussed and negotiated into a final set of themes. In step 3, the 3 authors performing the analysis created subthemes and abstracted the content of the individual meaningful entities into each subtheme in the condensates. In step 4, the meaning of the condensates was summarized. The themes and subthemes were reviewed and rearranged into a final structure by AGE, DN, and CV. The authors met regularly throughout the analysis process, considering different ways of interpreting the results, and reached consensus through discussions.

Finally, the themes were approved before the authors selected representative quotes to support the analysis. The full transcripts were not translated from Norwegian to English. However, the third author (LSN), who is bilingual (ie, Norwegian or English), was well familiar with the transcripts and ensured that the translation of quotes and other aspects needed for interpretation preserved the original meaning of the transcripts and quotes.

### Ethical Aspects

The study was planned and performed in compliance with the principles outlined in the Declaration of Helsinki [49]. The study was approved by the Regional Committee for Medical and Health Research Ethics (REK 2018/8911) and the Oslo University Hospital Department for Data Protection and Information Security (ie, institutional review board equivalent; PVO 2017/6697). Written informed consent was obtained from all participants.

To ensure anonymity and privacy of the participants, personal data (eg, contact information and date of birth) and signed consent forms were stored on a secure server at Oslo University Hospital, separately from the collected data from the interviews. Each participant was assigned a study identity number, and the codebook connecting this identity number to the participant was stored separately on the secure server. During the audio-to-text transcription process, all names were removed, the data were deidentified, and the analysis was conducted using the deidentified transcribed text only.

## Results

### Participant Information

A total of 6 patients were approached and agreed to participate in the study. Of the 6 participants, 1 (16.7%) did not complete the program because of personal life events and was therefore not interviewed after the intervention and 5 (83.3%; 4 women and 1 man) completed the program and participated in the interviews. These participants spent 10 to 15 weeks from the time they received access to the EPIO program until all the 9 modules were completed, and the interviews were conducted. During the same period, the participants had 7 to 13 PMP face-to-face sessions. The patients who completed the program were aged between 30 and 70 years, with an average age of 51 (median 52) years. All patients had lived with pain for more than 3 months, most of them for many years (ie, mean 12.4, range 2-40 years). Diagnoses included pain after trauma or postaccident traumas, neck pain, and diffuse pain in the musculoskeletal system. Other diagnoses included posttraumatic stress disorder, generalized anxiety disorder, and attention-deficit/hyperactivity disorder. All participants reported struggling with the impact of pain on various areas of their daily lives. The familiarity with the use of mobile apps varied from moderate to very experienced, with 4 of the patients completing the program reported having previously used other health-related mobile apps.

### Overview

**Figure 2:**
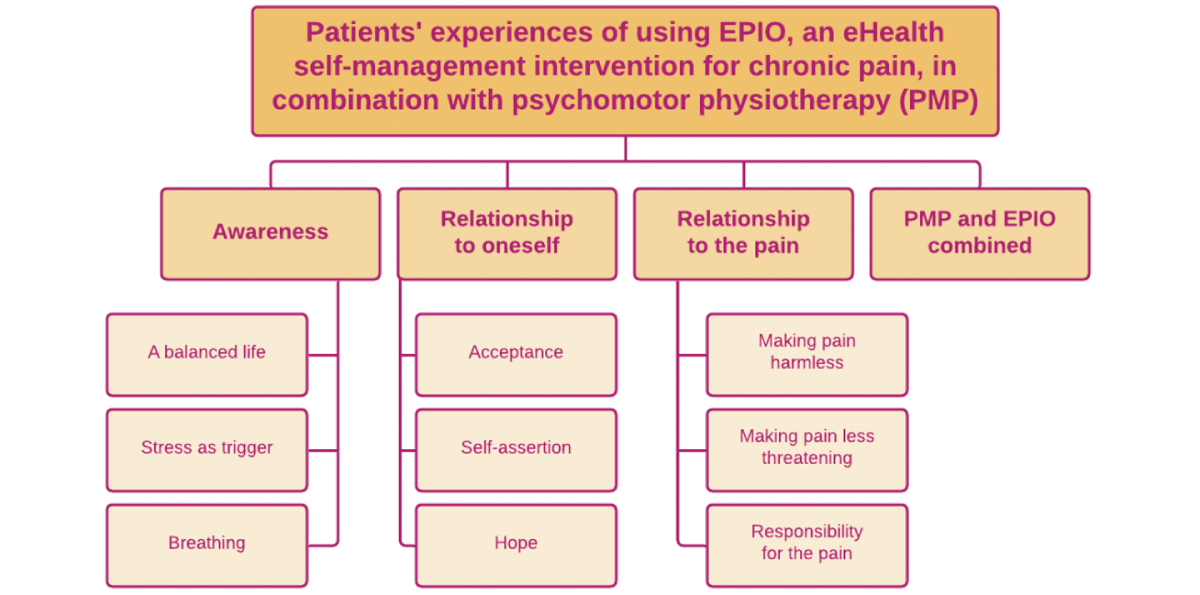
Overview of the main themes and subthemes. PMP: psychomotor physiotherapy.

Transcripts from the interviews were classified into four main themes: (1) awareness, (2) relationship to oneself, (3) relationship to pain, and (4) PMP and EPIO combined. The main themes and subthemes are illustrated in Figure 2.

### Awareness

#### Overview

All participants reported increased awareness about how they lived their lives and how they have become more attentive toward their body after using EPIO combined with PMP. The changes described related to awareness were sorted into three subthemes: a balanced life, stress as a trigger, and breathing. The participants described changes in how they noticed and reacted to signals from their body and described new thoughts about balance in life as a key to being able to live better with pain. All participants also reported increased awareness of the causes of stress and ways to better handle stress.

#### A Balanced Life

All participants described how EPIO had helped them to consider their own balance of rest and activity, and most of them stated that they, because of EPIO, had become aware of how their activity level was often higher than what they had thought. Some of the participants described becoming aware of a gap between how they wanted, and tried, to live their lives and the extent to which they were actually able to live their lives this way. Explaining how EPIO had helped, one participant stated the following:

I have been ‘on’ for as long as it is possible to be “on”. Until I’m exhausted, and then I am “off”. So, to keep it more even during the days, it is easier to remind myself now that I have this more at the forefront of my mind.

Participant 5

A number of the participants also stated that they had discovered how important it was to take short breaks and be conscious of their level of activity, rest, and sleep. Some reported feeling calmer after using EPIO, and some reported that they used EPIO as a reminder to take breaks and perform EPIO exercises throughout the day. A participant said this about how EPIO gave concrete suggestions to balance activity and rest:

But this was very specific, you know, can you do half the dishes and then rest? I never even thought of it that way.

Participant 4

#### Stress as a Trigger

All participants stated that the use of EPIO had made the relationship between stress and pain clearer to them and that they realized they had to do something about the stress and the way they dealt with stress in their lives. A participant said the following:

It was quite early [after being introduced to EPIO], that I had to realize the importance of stress-reducing approaches.

Participant 3

Participants did, however, present different stories about the causes and nature of their stress reactions. Some described stress at work or at home, whereas others described having been exposed to life trauma causing bodily and psychological stress. Several participants stated that EPIO helped them gain an increased awareness of the origin of their stress responses and had also provided tools for better self-management. A participant stated the following:

To realize that my breathing is connected to stress and traumas and improvement has been surprising. When I get flashbacks [...], this is what happens. My body goes into alert mode. [...] Sometimes it takes time to calm it down, sometimes it goes more quickly. But I HAVE a tool, to get my body out of alert mode. And it is the breathing techniques from the app.

Participant 4

Participants also described often finding it challenging to make the changes needed to lower their stress levels, stating that taking frequent breaks and doing breathing exercises or actively using approach coping strategies can be easier said than done during demanding or stressful times. A number of them stated that it may take time and effort to start using such strategies frequently and that such suggested changes and approaches may sometimes seem impossible when an actual stressful situation occurs. However, all participants described the face-to-face contact with, and follow-up by, the psychomotor physiotherapist as an advantage when aiming to make the necessary changes. One participant reported realizing their home situation was their main source of stress, with years of *serving* other family members making the participant feel as if having almost *disappeared* as an individual. For the road ahead, the participant shared the following:

Having insight, or taking time for yourself, it’s kind of...It hasn't really been there before, and it opens a few doors that can create a bit of a mess around you. Not just for yourself, but for the people around you too [...] Getting insight related to the fact that I do things for others all the time, for everyone else...It can make me irritated, and maybe a little bit angry [...] I haven't been angry for years. Maybe this is a good thing, there is after all a reason for all the tensions.

Participant 3

The quote illustrates how challenging, and even stressful, it can be for a person to make the necessary suggested changes and how it can also impact people around them and their relationship with them.

#### Breathing

All participants described experiencing becoming more aware of, and attentive to, their bodies through the use of EPIO. The focus on breathing and diaphragmatic breathing techniques in EPIO was reported to be among the most important parts of the program, with the breathing focus reportedly bringing on reflections about, and experiences of, how deep breathing could help make the mind as well as the body more relaxed. Some of the participants had very specific experiences of how breathing could impact their pain, and one stated the following:

It was shocking to realize how breathing specifically helps with tolerating and coping with pain. I’m thinking, it hurts, I will try using the EPIO app now. Then I do one of the exercises, and I notice, it hurts to breathe, but I can see that...When I do that exercise, and manage to breathe properly, the pain eases...It eases and becomes more tolerable.

Participant 4

Most participants described finding value in receiving information about the importance of deep breathing, stating that this created a new awareness about their own breathing and tension patterns. They also emphasized the importance of being guided through breathing and relaxation exercises by the EPIO voice, to feel the difference guidance could make, and the importance of being encouraged to repeat the exercises on their own over a period. With this new awareness related to the role of breathing, participants described the EPIO exercises as helpful in breaking old patterns. A participant stated the following:

My body can find relaxation without me forcing it to. I don’t have to force my shoulders to relax in order to breathe. I can just think about it, and balance occurs. It has strengthened my belief, or convinced me, that things are connected. There is no doubt. Our mind controls a lot of what we do, whether we are aware of it or not. The fact that you can actually lead yourself out of what has just occurred - without even really being aware.

Participant 1

### Relationship to Oneself

The participants reported that EPIO had contributed to changes in how they related to themselves, with some of them even referring to EPIO as a program for self-management of life in general, sometimes even forgetting that the program was about pain and pain management. The changes described as experienced by the participants were sorted into three subthemes: acceptance, self-assertion, and hope.

#### Acceptance

Some participants described that EPIO had helped them gain greater self-acceptance, stating that they now felt more confident, with a stronger belief in themselves, realizing that they were not *a bad person*, even if the pain kept them from doing and managing things the way they had previously been able to. A participant stated the following:

Some of the things I have learned using EPIO have made it easier for me to accept life the way it is. And to understand it, and not beat myself up about it.

Participant 1

In addition, some of the participants found it important to consider the qualities and skills they still had, what they wished for in life, and what they appreciated in the people they were close to. A participant said the following:

And, I think, it’s not like I have to reinvent myself. [...] No, I AM there! [...] So the value, my personality, is not gone. The skills are there, but I cannot do as much as I did before.

Participant 4

#### Self-assertion

Several of the participants described feeling empowered by some of the content in the EPIO program, stating that EPIO encouraged them to assert themselves. Some of the participants described finding time to do what was important and right for themselves as challenging and unfamiliar, stating that prioritizing themselves and asking for help appeared unfamiliar and had not had precedence in their lives. A participant said the following:

I am the person helping others. No one thinks I should need help. So I really have myself to thank for that. People do not come and ask.

Participant 5

However, some of the participants described appreciating being reminded of the importance of taking time for themselves, prioritizing their own health, wishes, and needs. A participant even related his or her pain to the lack of prioritizing themselves and stated the following:

The pain in my muscles, it can of course come from the fact that I live to help others all the time, and forget to help myself in a way. And that’s in this program. Help with self-management.

Participant 3

#### Hope

Some of the participants also reported having obtained new hope in life from the use of EPIO in combination with PMP. They described having gained hope of being able to manage life in a new and different way, including hope of being able to live a more balanced life, to know and actively use coping strategies in day-to-day life, and through that being able to live life, even with pain—in a more fulfilling and *normal* way. They described being able to have a tool such as EPIO, accessible anywhere and anytime, as providing them hope for better quality of life and a better future ahead. A participant described the link between EPIO and new hope this way:

This program has given me hope that I can continue to work on this. I will have a life with quality, I will have a life I can manage. I will live a life I don't have to be ashamed of. I will be a complete person, but I will do things a little differently, and at a different pace.

Participant 4

### Relationship to the Pain

The participants described a number of ways that EPIO had contributed to changes in how they related to their pain. Descriptions of such changes related to experiences that EPIO had contributed to making the pain less threatening, encouraged and stimulated the participants to use distractions from the pain, and changed their view of their own responsibility in terms of managing the pain.

#### Making Pain Less Threatening

Most participants reported that they, after starting to use the EPIO program, had experienced less pain-related anxiety and described EPIO as helpful in making the pain seem less threatening in their day-to-day life. To some extent, it had been important to gain new insights into physiological explanations and pain coping techniques. They stated that the information and knowledge gained through EPIO made it easier to relate to potential causes of pain and understand the relationships between pain, thoughts, and feelings and that the information gained through EPIO made it easier to believe that the pain might not necessarily be dangerous. Learning new coping strategies also appeared to have contributed to making the pain seem less threatening, as expressed by a participant:

I can cope better with my pain if I know I have techniques I can use during the day to make it a little less tense and painful. Yes, the pain is still there, but…It does not get as much attention.

Participant 2

#### Distraction From the Pain

The EPIO program contains a number of coping strategies and exercises related to distraction from the pain, and all participants in the study described learning and using these techniques, aiming to distract and remove the focus from pain, as helpful. Some of the participants even explained that because of deliberately using these distraction techniques, they now had periods where the pain appeared to absorb less of their day-to-day attention. Others stated that the pain was still present but that it might be less dominating. One of the participants described the increased ability to distract themselves from the pain as follows:

Just to be able to move away from focusing on the pain. That things in my body are not as they should, to just focus on something else for a while. A break. [...] To just notice what happens around you. Not just inside you. Focus on that.

Participant 2

#### Responsibility for the Pain

After using EPIO, several participants stated that discovering strategies to influence and cope with their pain had made them realize that they have a greater responsibility of managing their own pain than what they had previously thought. They all stated that they actively had to handle the pain and described how they could see more clearly how their pain related to the way they lived their lives, including impact on stress factors, relationships, breathing habits, and bodily tensions. However, most participants stated that making the necessary changes in life would be energy consuming and that establishing lasting changes would require motivation and energy. One participant described making significant changes as “impossible” because of the situation at home. Another participant described the significant work required to obtain the effect as follows:

Pain is something I have to relate to in one way or another. How I think is very important, and I have to think that I can handle things in a different way. Quick fix, that is what you want [...] And this is the opposite, in a way. You have to really delve into it, absorb it, and let it have consequences for your body.

Participant 1

However, the same participant added a contrary reflection, noting the possibility that EPIO could potentially produce guilt and feelings of resignation should the user do everything to integrate what they had learned while using the app, without experiencing improvements in pain levels:

I have felt it myself and, well now I have felt better, and it may be because I have done all the right things. And if you’re then suddenly in pain, then it may feel like it's your own fault.

Participant 1

### PMP and EPIO Combined

All participants in this study reported experiencing EPIO and PMP as complementary and stated that they found it easier to perform exercises and focus on pain management strategies while using EPIO in combination with PMP treatment. All participants described EPIO as a reinforcing tool for their pain self-management, bringing new aspects and themes to their PMP treatment, and reported finding it easier to work on their own, in between PMP treatment sessions, with EPIO at hand. They described EPIO as an enforcer, making it easier to integrate the necessary pain self-management into daily life. One of the participants said the following:

I think this [EPIO] is a very useful tool to use in parallel [with PMP], and the patient can work in between. I think it could help many to integrate it into day-to-day life.

Participant 2

Several participants also pointed to the combination of self-management through EPIO and face-to-face or physical touch provided by PMP as an important part of the process of raising mental and physical awareness. All participants described the massage techniques learned from PMP as having helped them become more aware of tension in their muscles, making it easier to achieve relaxation. One of the participants, having experienced significant traumas in life, also stated that the combination of PMP and EPIO helped involve the body as well as the mind in the process of treatment and healing.

During the interviews, participants frequently mentioned how making changes can be difficult and sometimes even frightening. As such, having a combination of in-person or EPIO treatment available was seen as a strength, enabling learning at the participants’ own pace but with the safety of in-person discussions and guidance when needed. A participant stated the following:

I think you need a combination. And some time. Because this is something you can choose not to do. With everything else going on in life. It's easier to just sit down and read the paper. And if you are in great pain, you don't have that much to go on.

Participant 5

As EPIO was described as comprehensive, all participants stated that it was helpful to work together with the psychomotor physiotherapist to work through potential issues that occurred and sort out what was the most important aspect to focus on between each consultation. Several patients also stated that EPIO had started a process in which they had to actively keep working. They all said that they thought EPIO alone would have brought new experiences also without PMP but that the combination strengthened the impact.

## Discussion

### Principal Findings

This study explored patients’ experiences of using the app-based intervention EPIO in combination with PMP for self-management of chronic pain. The study participants described experiencing EPIO as bringing well-known as well as new themes into the treatment and as supporting self-management between face-to-face sessions. Following the combined EPIO and PMP program, participants described increased awareness of a number of issues, including the importance of balancing rest and activity, the relationship between stress and pain, and the importance of being aware of signals from their bodies. They also reported that EPIO had contributed to changes in their relation to themselves, including increased self-acceptance and self-assertion, willingness to prioritize themselves, and increased hope for a better life. Participants’ relationship with the pain itself was also reported to have changed when using EPIO in combination with PMP, including experiencing pain as less threatening, benefiting from the use of distractions, and taking more responsibility for self-management of the pain.

### Comparison With Previous Work

Findings show that the participants experienced living and coping with pain as complex—involving challenges related to many aspects of life, including stress. Some researchers have suggested a link between stress and hypersensitivity to pain [50,51], and in accordance with existing research [52], participants in this study described how pain was triggered and reinforced by psychosocial stressors. The introduction of EPIO into PMP treatment led to positive changes in how the participants perceived their pain and approached their daily lives. In line with studies showing the interrelation between stress, pain, and coping [51,52], participants described becoming more aware of causes of stress in their daily lives, and how stress affected them and triggered their pain. Through the blended treatment of EPIO and PMP, most participants reported having found new strategies to deal with life stress and better manage their pain.

The participants described psychosocial stressors related to social relationship challenges, feelings of guilt, difficulties meeting the many demands of daily life, and a general feeling of not being enough and constantly falling short of demands and expectations. Research has suggested that people living with chronic pain may benefit from treatment approaches aimed at reducing feelings of guilt, finding ways to cope with the many demands, and increasing levels of confidence [53]. Participants in this study reported becoming more self-accepting, assertive, and confident and feeling less shameful and vulnerable, which may therefore be of importance in this setting.

The interrelationship between fear, anxiety, and pain is commonly reported among patients with chronic pain, and scholars emphasize the importance of addressing these aspects in treatment [54,55]. In this study, participants reported having become less fearful and anxious about their pain following the blended EPIO and PMP treatment, attributing the changes to the general information about pain and tools for better coping provided by EPIO.

Following the blended EPIO and PMP treatment, participants in this study described having become more aware of breathing in general and the importance of deep breathing in particular. The importance of breathing is a core part of PMP treatment and had already been introduced to participants before the study. Still, some exercises in the EPIO program reportedly provided new experiences regarding the relationship between deep breathing and pain management, apparently raising new awareness of these relationships, and some of the participants reported experiencing reduced levels of stress and pain because of practicing breathing technique exercises. As deep, diaphragmatic breathing can impact breathing frequency (ie, slowing), these findings can also support the notion that associations exist between breathing frequency and pain [56].

Participants described a new awareness of the relationship between rest and activity. Some reported realizing that their activity level had reached beyond their capacity, and that this had been the case for quite some time. These participants described it as difficult to take breaks during the day and ask for assistance. Their observation of the importance of a better balance between rest and activity corresponds well with research reporting that imbalance can lead to reduced pain tolerance and decreased quality of life [57,58].

Self-management tools such as EPIO generally aim to help patients take an active role in managing their pain and day-to-day life. Self-efficacy is often seen as a determinant for keeping up efforts and not giving in when faced with obstructions [59], and findings from this study may indicate an increased sense of self-efficacy among participants posttreatment, in terms of a strengthened belief in being able to live a life less impacted by pain. A strengthened sense of self-efficacy may also have helped them make the necessary changes in life to achieve better coping.

Self-management can be demanding, entailing taking responsibility to *manage* one’s own life, maintaining good habits, and making changes when needed. To make the necessary changes requires energy and motivation, of which the patient may already be drained or depleted because of the many demands involved in living with chronic pain [1,12,60]. Although participants reported gaining new awareness and knowledge related to pain and self-management of pain from the EPIO program, they all underlined the demands and costs of being the agent of self-management and change in this way. EPIO was not considered *a quick fix* either, as the program requires users to be active and engaged throughout. Self-management programs such as EPIO may help users discover what they can or need to do to better live *with* pain. However, if unable to undertake the necessary steps toward better self-management, knowing that life could be better *if only* changes are made may possibly also produce feelings of guilt and shame, making life even worse. This implies responsibility and developers and/or providers of such self-management programs should be aware of the potential ethical implications of creating and recommending such independent self-management programs, regardless of potential benefits and intent. Participants in this study described appreciating the combination of EPIO and PMP in their treatment, and it could be that self-management programs such as EPIO could best be recommended in combination with existing care and in-person follow-up, as also supported by the participants in this study. However, several participants reported having gained increased awareness of their own responsibility for self-management in this study, which they were also willing to take. It is possible that, despite the demanding aspects of self-management, the CBT and ACT basis of EPIO may have helped the participants achieve a changed relation to their pain, pointing them toward a greater acceptance of living with pain and an enhanced knowledge of what could be done.

The use of blended care, here referred to as combining in-person and eHealth programs, may have a positive impact as patients may benefit from the best of 2 or more treatment approaches [38,39]. Having EPIO as a tool to work with between PMP sessions appeared to help the participants in this study, and this type of blended care may be suitable for stress- and pain-related self-management and coping, as indicated in this study.

With more than one treatment option available (eg, PMP and EPIO), patients can have the option to decide for themselves how to meet their treatment needs, for example, from their psychomotor physiotherapist or from a program such as EPIO or both. In line with previous research, the results from this study indicate that many prefer to go through the eHealth program at their chosen pace [61], without a health care provider or coach and guide trying to impact their progress. Furthermore, compared with in-person interventions, eHealth interventions can be experienced as a more neutral and nonjudgmental form of delivery, eliminating the fear of being judged or misunderstood by a provider [61]. This corresponds with participants’ description of finding it easier to go through the contents of EPIO on their own, without necessarily discussing everything with the psychomotor physiotherapist. At the same time, some of the participants brought relevant topics from their EPIO use into psychomotor therapy; topics they may not have discovered had the information been less comprehensive.

Another important reason for combining EPIO and PMP is the notion that the patient–provider relationship can affect patient outcomes. A strong therapeutic alliance between the patient and health care provider can potentially predict positive treatment outcomes [62,63]. In addition, speaking to another person can be helpful. Simultaneously, if the therapeutic alliance is weak, the treatment process may become more difficult. In such cases, an option such as EPIO can be particularly useful. eHealth can likely not replace the relational processes involved in in-person treatment. However, eHealth interventions can offer support when in-person treatment is not available, and at the very least, eHealth can be offered as a supplement to in-person treatment and care, as in this study. Through EPIO, the participants in this study received a CBT and ACT approach beyond PMP regular competence, describing the information and content in EPIO as meaningful and complementary to PMP treatment.

### Strengths and Limitations

This study has a number of limitations. First, the study was conducted at a single physiotherapy institute and included a limited number of participants. However, although the sample was small, the data material represented richness and depth about how the participants experienced using EPIO in combination with PMP, and statements from one participant could be as important as statements of most participants. Given the small predefined number of participants in the study, all participants were identified and selected by the psychomotor physiotherapist to ensure that the participants could use EPIO in the study period and be able to attend a postintervention interview. This could have introduced a selection bias and as such be considered a limitation, but it also ensured professional soundness (ie, able to attend the study). Although conducted with a limited number of participants, the interviews conducted in this study provided insight into a variety of patient experiences and allowed sufficient depth in the analyses, which can be considered a strength.

Second, 80% (4/5) women and 20% (1/5) men participated in this study. The results of the study might have been different if more men had participated. For example, men might bring different experiences of using EPIO in combination with PMP. However, the gender distribution in the study represents the general gender distribution among patients receiving PMP, and additional men were not available for inclusion at the time the study was conducted. This is also in line with the first author’s experience from clinical practice, with approximately 1 man per 12 to 15 women enrolled to receive PMP. In addition, given the range related to age, years living with pain and other diagnoses, and most women living with chronic pain, the study can be considered representative of patients living with chronic pain. In addition, with the rich data material collected during and following study participation, even a small number of participants allows for in-depth insight related to participants’ experiences using EPIO in combination with PMP.

As 80% (4/5) participants in the study had previously used health-related mobile apps, a certain bias related to previous eHealth experience could be present in the current findings. Such experiences could impact participants’ opinions and views, and perceived benefits and findings might have looked different if the sample included a more balanced sample of experienced or inexperienced users. Experience in using digital tools could, however, impact findings positively (eg, have enjoyed using mobile apps) and negatively (eg, did not like using mobile apps), and the outcome of having a more balanced sample may therefore be challenging to predict.

Participants were also identified and selected by the first author and were already enrolled in the first author’s PMP treatment program. This may have implied a selection bias but may also have ensured professional soundness (ie, patients who were able to complete the EPIO program). To ensure that patients did not feel coerced to participate in the study, they received oral and written information stating that study participation was voluntary; nonparticipation would have no consequences for their ongoing PMP treatment; and they, should they decide to participate, could withdraw from the study at any time without having to give a reason. Patients received information about the study in one PMP session and could then wait until a later time point (ie, time to reflect) to decide whether to participate and give their written consent. To limit the risk that participants provided more positive than negative feedback because of their relationship with the psychomotor physiotherapist, the participants were informed that study results would not affect the psychomotor physiotherapist, the ownership of EPIO was elsewhere, and the psychomotor physiotherapist did not receive any benefits or have any negative consequences, regardless of the study findings. Before the study interviews, participants were also informed that to truly evaluate the program, their honest feedback on EPIO, and about using EPIO in combination with PMP, was needed.

Throughout the study process, the authors adopted a reflexive approach to the dual role of the researcher or psychomotor physiotherapist. This was a point of discussion in all research meetings throughout the process and was also addressed by closely involving two of the coauthors in the data analysis process.

Third, the first author’s professional background as a psychomotor physiotherapist could have influenced the process (ie, from interviews to manuscript write-up) through preconceptions drawing attention to certain aspects of information. The involvement of 2 coresearchers during analysis was, however, actively used to counter such threats to the trustworthiness of the findings [64].

Finally, the results showed that living with pain is overly complex, and a small qualitative study such as this cannot provide information about what contributed the most to participants’ experienced benefits of using EPIO or the combination of EPIO and PMP. As such, factors unrelated to the EPIO tool may have contributed, including regular follow-up by a health care provider and the ability to talk about EPIO at every consultation. The close follow-up may also have contributed to the participants making a greater effort with EPIO than those who did not have the blended care type follow-up. The qualitative in-depth insight provided, even with a small sample, is a major strength of this study.

### Conclusions

This study offers insight into chronic pain patients’ experiences with the use of EPIO, an app-based intervention for the self-management of chronic pain, in combination with PMP. Findings indicate that EPIO, used in this blended care context, was experienced as beneficial in raising awareness and finding a better balance between rest and activity; feeling more valuable as a person; and increasing self-acceptance, self-assertion, and hope and in relation to the participants’ pain—seeing pain as less harmful and engaging in more active stress management and coping strategies. In conclusion, this study indicates that a blended care approach combining eHealth self-management interventions such as EPIO with PMP may be of value to patients living with chronic pain.

## Appendix

Multimedia Appendix 1 Interview guide.

## Abbreviations

ACT: acceptance and commitment therapy
CBT: cognitive behavioral therapy
PMP: psychomotor physiotherapy
