# Supplementary material for: Patients’ Experiences of Using an eHealth Pain Management Intervention Combined With Psychomotor Physiotherapy: Qualitative Study
Source: JMIR Form Res. 2022 Mar 16;6(3):e34458. doi: 10.2196/34458 (PMC8968559; doi:10.2196/34458)
Supplement: Multimedia Appendix 1 [file formative_v6i3e34458_app1.docx]

Multimedia Appendix 1

# Interview guide

What are your experiences using EPIO? Is EPIO something you would recommend others to use? Why, why not? How did you use the content of the application? What was important to you? What was less important?

Has EPIO influenced what it's like to live with your pain? In what way? Has EPIO led to using any new coping strategies? Which ones, in which case?

What has EPIO done for you and how?

The combination of psychomotor therapy and EPIO, can you say anything about how you experienced it?

Has EPIO initiated any thoughts about raising awareness of other parts of your life that don't directly target pain?

Are there any prerequisites for you to make EPIO useful? Prerequisites in relation to surroundings and other help and support? Something about the app that could have been different to increase its usefulness? Using an app in itself, what do you think of it?
